# Circulating Lipid Traits and Ovarian Cancer Risk: A Systematic Review and Meta-Analysis with Mendelian Randomization Integration

**DOI:** 10.3390/metabo16050290

**Published:** 2026-04-23

**Authors:** Marco Marian, Andrei Ardelean, Mihai Rosu, Cristi Tarta, Alexandru Isaic, Dan Brebu, Camelia Marian, Ioana Adelina Faur, Paul Pasca, Ionut Flaviu Faur, Dana Stoian, Andrei Korodi

**Affiliations:** 1 Doctoral School, Victor Babes University of Medicine and Pharmacy, E. Murgu Square, No. 2, 300041 Timisoara, Romania; marian.marco@umft.ro (M.M.); adelina.clim@umft.ro (I.A.F.); paul.pasca@umft.ro (P.P.); 2 Researching Future Surgery II Research Center, Department X, Discipline of General Surgery II, Faculty of Medicine, Victor Babes University of Medicine and Pharmacy, E. Murgu Square, No. 2, 300041 Timisoara, Romania; tarta.cristi@umft.ro (C.T.); isaic.alexandru@umft.ro (A.I.); brebu.dan@umft.ro (D.B.); camelia.cioenaru@rezident.umft.ro (C.M.); 3 1st Surgery Clinic, Emergency Clinical County Hospital of Arad, 2-4 Andreny Karoly Str., 310037 Arad, Romania; rosu.mihai@uvvg.ro; 4 Center of Molecular Research in Nephrology and Vascular Disease, Faculty of Medicine, Victor Babes University of Medicine and Pharmacy, E. Murgu Square, No. 2, 300041 Timisoara, Romania; stoian.dana@umft.ro; 5 2nd Department of Internal Medicine, Victor Babes University of Medicine and Pharmacy, E. Murgu Square, No. 2, 300041 Timisoara, Romania; 6 2nd Surgery Clinic, Emergency Clinical County Hospital of Arad, 2-4 Andreny Karoly Str., 310037 Arad, Romania; korodi.andrei@uvvg.ro; 7 Department of Medicine, Faculty of Medicine, “Vasile Goldis” Western University of Arad, L. Rebreanu St. 86, 310048 Arad, Romania

**Keywords:** ovarian cancer, triglycerides, HDL cholesterol, lipid metabolism, meta-analysis, Mendelian randomization, metabolic risk factors, cancer epidemiology

## Abstract

**Background**: Metabolic dysregulation is increasingly recognized as a contributor to carcinogenesis; however, the role of circulating lipid traits in ovarian cancer remains unclear. **Methods**: A systematic review and meta-analysis were conducted following PRISMA 2020 guidelines. PubMed, Web of Science, Scopus, and Embase were searched from inception to March 2026. Observational studies evaluating triglycerides (TG), high-density lipoprotein cholesterol (HDL-C), low-density lipoprotein cholesterol (LDL-C), and total cholesterol (TC) in relation to ovarian cancer risk were included. Random-effects models were used to pool relative risks (RRs). Robustness was assessed via sensitivity analyses, influence diagnostics, and multiverse analysis. Mendelian randomization (MR) evidence was integrated for causal inference. **Results**: Six observational studies were included in the meta-analysis. Elevated triglyceride levels were associated with increased ovarian cancer risk, while HDL-C showed a modest inverse association. LDL-C and total cholesterol were not significantly associated with risk. Sensitivity analyses excluding early follow-up strengthened the triglyceride association. MR analyses supported a potential causal role for triglycerides but not for HDL-C. **Conclusions**: Circulating triglycerides may represent a metabolically relevant risk factor for ovarian cancer. Further large-scale prospective and mechanistic studies are warranted.

## 1. Introduction

Lipids constitute essential components of cellular physiology, playing critical roles in membrane biogenesis, energy storage, and intracellular signaling cascades [1,2,3,4,5,6]. In the context of malignancy, cancer cells undergo profound metabolic reprogramming, frequently characterized by a shift toward a lipogenic phenotype, which enables them to sustain the energetic and biosynthetic demands of uncontrolled proliferation [7,8,9,10,11,12,13,14,15,16]. This metabolic adaptation encompasses increased uptake of exogenous lipids, upregulation of de novo lipogenesis, and enhanced utilization of fatty acid oxidation pathways [17,18,19,20,21,22,23]. Collectively, these processes support membrane synthesis, energy production, and the generation of lipid-derived signaling molecules, thereby facilitating tumor growth and survival [24,25,26,27,28,29]. Consequently, systemic lipid metabolism and circulating lipid levels may critically influence the metabolic milieu that promotes tumor initiation and progression [30,31,32,33].

Among circulating lipid fractions, triglycerides (TG), high-density lipoprotein cholesterol (HDL), low-density lipoprotein cholesterol (LDL), and total cholesterol (TC) represent routinely measured biomarkers in clinical and epidemiological settings. Triglycerides, as the principal storage form of fatty acids, constitute a major source of metabolic fuel that can be mobilized by tumor cells [34]. Elevated circulating triglyceride levels are frequently associated with metabolic syndrome, insulin resistance, and chronic low-grade inflammation—conditions that have been increasingly implicated in carcinogenesis through mechanisms involving hyperinsulinemia, oxidative stress, and pro-inflammatory signaling pathways [35,36]. In contrast, HDL cholesterol exerts a range of protective biological functions, including reverse cholesterol transport, antioxidative activity, and modulation of inflammatory responses, which may mitigate cellular lipid accumulation and oxidative damage, thereby potentially exerting anti-tumorigenic effects [37]. Despite the biological plausibility of these mechanisms, epidemiological evidence examining the relationship between circulating lipid traits and ovarian cancer risk remains inconsistent. Several studies have reported positive associations between elevated triglyceride levels and ovarian cancer incidence, suggesting a potential role of dysregulated lipid metabolism in tumor development [38,39,40,41]. Conversely, HDL cholesterol has been associated with reduced cancer risk in some studies, while others have reported null findings. For LDL cholesterol and total cholesterol, the available evidence is particularly heterogeneous, with most studies demonstrating weak or inconsistent associations. Such discrepancies may reflect variations in study design, population characteristics, timing of lipid measurement, and the extent of adjustment for metabolic and lifestyle confounders [42,43,44].

Moreover, the interpretation of observational associations between lipid traits and cancer risk is inherently limited by the potential for reverse causation and residual confounding, especially given that metabolic alterations may precede clinical diagnosis during the preclinical phase of disease [45,46,47]. In this context, Mendelian randomization (MR) has emerged as a powerful analytical framework to strengthen causal inference in epidemiological research. By leveraging genetic variants associated with lipid levels as instrumental variables, MR analyses can provide estimates that are less susceptible to confounding and reverse causation, thereby offering complementary insights into the potential causal role of lipid metabolism in ovarian carcinogenesis [48,49,50,51]. Although prior studies and reviews have explored the relationship between lipid metabolism and ovarian cancer, a comprehensive synthesis integrating observational evidence with advanced methodological approaches remains lacking. In particular, few investigations have systematically evaluated the robustness of pooled estimates across alternative analytical specifications or assessed the influence of individual studies on overall conclusions [52,53,54].

Therefore, the present study aimed to conduct a comprehensive systematic review and meta-analysis examining the association between circulating lipid traits and ovarian cancer risk. Specifically, we evaluated the relationships between triglycerides, HDL cholesterol, LDL cholesterol, and total cholesterol and the incidence of ovarian cancer. Beyond conventional meta-analytic techniques, we incorporated a range of advanced analytical strategies—including cumulative meta-analysis, influence diagnostics, specification curve (multiverse) analysis, and exploratory assessments of publication bias—to rigorously assess the stability and robustness of the findings. Furthermore, we integrated observational results with Mendelian randomization evidence through a triangulation framework to enhance causal interpretation. By combining epidemiological evidence with modern analytical methodologies, this study seeks to provide a more nuanced and comprehensive understanding of the role of systemic lipid metabolism in ovarian cancer development, and to identify metabolic pathways that may represent promising targets for future research in cancer prevention and mechanistic investigation.

## 2. Material and Methods

### 2.1. Study Design and Reporting Standards

This systematic review and meta-analysis was conducted in accordance with the PRISMA 2020 guidelines. The analytical framework combined conventional meta-analytic approaches with advanced robustness and causal inference techniques, including cumulative meta-analysis, influence diagnostics, trim-and-fill assessment, specification curve (multiverse) analysis, and triangulation with Mendelian randomization (MR) evidence.

### 2.2. Literature Search Strategy

A comprehensive search was performed in PubMed/MEDLINE, Web of Science, Scopus, and Embase from database inception to the final search date. The search strategy combined terms related to lipid metabolism and ovarian cancer using Boolean operators: (“triglycerides” OR “HDL cholesterol” OR “LDL cholesterol” OR “total cholesterol” OR “lipid profile” OR “circulating lipids”) AND (“ovarian cancer” OR “ovarian neoplasms”) AND (“cohort” OR “case–control” OR “prospective”).

### 2.3. Eligibility Criteria

Studies were included if they met the following criteria:

(i) Observational design involving adult women;
(ii) Assessment of circulating lipid traits (TG, HDL, LDL, or TC);
(iii) Outcome defined as incident ovarian cancer;
(iv) Reporting of effect estimates (RR, OR, or HR) with 95% confidence intervals;
(v) Comparison between highest versus lowest exposure categories or data allowing harmonization.

When multiple publications derived from the same cohort were available, the study with the largest sample size or longest follow-up was included.

### 2.4. Data Extraction

Two investigators independently extracted data on study characteristics (author, year, design, region), sample size and number of cases, lipid biomarkers, exposure categories, effect estimates with 95% CIs, covariate adjustment, and follow-up duration (for cohort studies). Discrepancies were resolved by consensus.

### 2.5. Statistical Analysis

#### 2.5.1. Meta-Analysis Model

Random-effects models (DerSimonian–Laird) were used. Heterogeneity was assessed using I^2^ statistics. Sensitivity analyses included restriction to prospective studies and exclusion of early follow-up. To address potential reverse causation, analyses excluded cases occurring within 2 years of lipid measurement. Alternative lag periods (e.g., 1-, 3-, or 5-year exclusions) were not systematically evaluated and represent an area for future research.

Where reported, subgroup analyses were conducted according to menopausal status (pre- vs. postmenopausal). Given variability in reporting and limited stratified data, these analyses were considered exploratory and interpreted with caution.

#### 2.5.2. Subgroup Analyses

Prespecified subgroup analyses were conducted according to:

- Histological subtype (serous vs. non-serous);
- Menopausal status (pre- vs. postmenopausal);
- Disease stage (early vs. advanced);
- Study design (prospective vs. case–control).

#### 2.5.3. Sensitivity Analyses

Robustness of the findings was evaluated through:

- Restriction to prospective cohort studies;
- Exclusion of studies with <2 years follow-up (to reduce reverse causation);
- Leave-one-out analyses to assess the influence of individual studies.

##### Influence Diagnostics

Influence diagnostics included:

- Baujat plots (heterogeneity vs. influence);
- Standardized residuals (outlier detection);
- Influence heatmaps, integrating study weight, contribution to heterogeneity, and leave-one-out impact.

#### 2.5.4. Cumulative Meta-Analysis

Cumulative meta-analyses were conducted for triglycerides and HDL by sequentially adding studies according to publication year, allowing assessment of temporal stability and evolution of pooled estimates.

##### Publication Bias Assessment

Publication bias and small-study effects were evaluated using funnel plots and Egger’s regression test. Additionally, trim-and-fill analyses were performed as exploratory assessments of the potential impact of missing studies.

#### 2.5.5. Specification Curve (Multiverse) Analysis

A multiverse analysis was conducted to assess robustness across alternative analytical specifications, including:

- Fixed-effect vs. random-effects models;
- Hartung–Knapp adjustment;
- Restriction to prospective studies;
- Exclusion of less precise studies;
- Leave-one-out scenarios.

Results were visualized using specification curves to evaluate the consistency of pooled estimates across analytical choices.

##### Mendelian Randomization Integration and Triangulation

To strengthen causal inference, observational findings were triangulated with MR evidence evaluating genetically predicted lipid levels. Triangulation plots compared pooled observational estimates with MR-derived causal estimates, providing complementary insights into potential causal relationships.

### 2.6. Statistical Software

All analyses were performed using Python-based statistical tools, with graphical outputs generated using Matplotlib. All tests were two-sided, and *p* < 0.05 was considered statistically significant.

## 3. Results

A total of 42 records were identified through database searching in PubMed, Web of Science, Scopus, and Embase. After removal of duplicates, 36 records remained for title and abstract screening. Twenty records were excluded at this stage based on predefined eligibility criteria. Sixteen full-text articles were assessed for eligibility, of which eight were excluded due to insufficient extractable effect estimates, overlapping study populations, inappropriate exposure definitions, or non-observational study designs. Ultimately, eight studies were included in the qualitative synthesis, and six observational studies were incorporated into the quantitative meta-analysis. Additionally, one Mendelian randomization study was included as part of the causal triangulation analysis.

**Figure 1:**
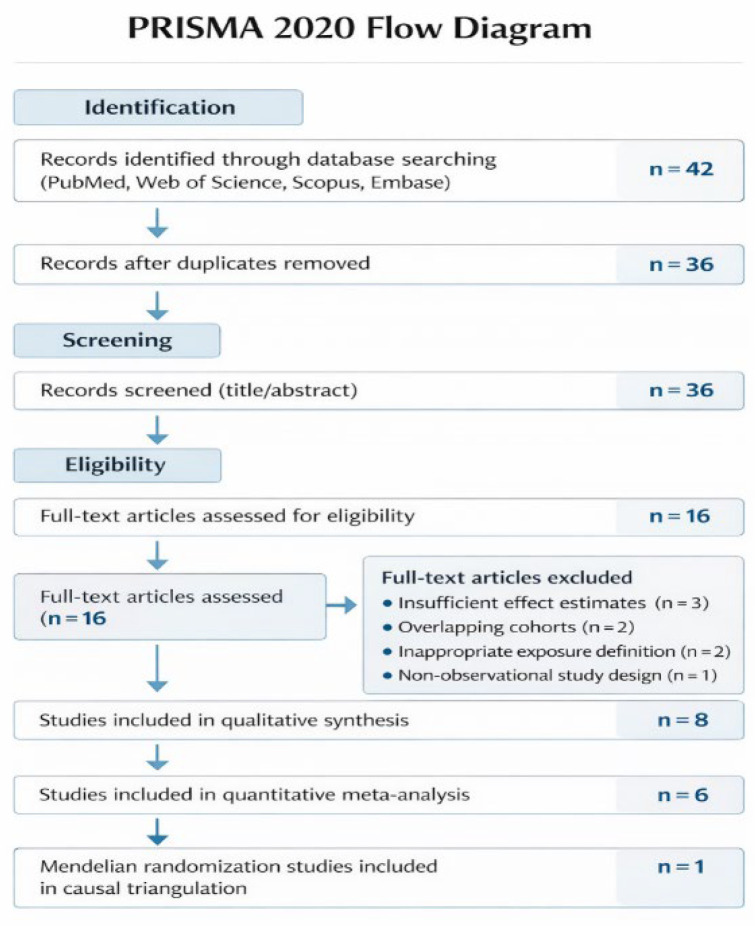
PRISMA 2020 flow diagram illustrating the study selection process.

The study selection process is summarized in the PRISMA 2020 flow diagram (Figure 1). The initial literature search across PubMed, Web of Science, Scopus, and Embase identified a total of 42 records. After removal of duplicate entries, 36 records remained and were screened based on title and abstract. During the screening phase, 20 records were excluded because they did not meet the predefined inclusion criteria, including studies not examining circulating lipid traits, studies focusing on non-ovarian cancer outcomes, and non-original research articles. A total of 16 full-text articles were subsequently assessed for eligibility. Of these, eight studies were excluded after detailed evaluation, primarily due to insufficient data for effect size extraction, overlapping study populations, inappropriate exposure definitions, or non-observational study designs. Ultimately, eight studies met the inclusion criteria and were included in the qualitative synthesis. Among these, six observational studies provided sufficient data to be included in the quantitative meta-analysis comparing the highest versus lowest categories of circulating lipid traits. In addition, one Mendelian randomization study was incorporated as a predefined causal evidence stratum to complement the observational findings.

The main characteristics of the studies included in the systematic review and meta-analysis are summarized in Table 1. The included studies comprised both prospective cohort and case–control designs and were conducted across diverse geographic regions, including North America, Europe, and Asia. Together, these studies included large population samples with varying numbers of incident ovarian cancer cases. The studies investigated associations between circulating lipid traits—primarily triglycerides (TG), high-density lipoprotein cholesterol (HDL-C), low-density lipoprotein cholesterol (LDL-C), and total cholesterol (TC)—and ovarian cancer risk. Exposure assessment was generally based on baseline measurements of serum lipid concentrations, which were categorized into quantiles (e.g., quartiles or tertiles) or clinically defined thresholds.

Most studies reported effect estimates comparing the highest versus lowest exposure categories, expressed as relative risks (RRs), hazard ratios (HRs), or odds ratios (ORs), with corresponding 95% confidence intervals. The majority of studies adjusted for key potential confounders, including age, body mass index (BMI), smoking status, reproductive factors, and in some cases hormone therapy use. Follow-up duration in prospective cohort studies ranged from several years to more than a decade, allowing assessment of long-term associations between lipid levels and ovarian cancer incidence. Overall, the included studies provided a diverse yet complementary body of observational evidence examining the potential role of circulating lipid traits in ovarian cancer risk.

The risk of bias of the included observational studies was evaluated using the ROBINS-I (Risk Of Bias In Non-randomized Studies of Interventions) tool, and the results are summarized in Table 2.

Overall, most studies were judged to have moderate risk of bias, primarily due to potential residual confounding and limitations inherent to observational study designs. Confounding was considered a key domain of concern, as lipid levels are strongly associated with metabolic factors such as obesity, insulin resistance, and lifestyle behaviors, which may also influence cancer risk. The risk of bias related to participant selection was generally low to moderate, particularly in prospective cohort studies where lipid measurements were obtained prior to cancer diagnosis. Exposure classification was considered largely reliable because lipid concentrations were measured using standardized biochemical assays in clinical or laboratory settings.

Missing data were reported to be minimal in most studies, and outcome assessment was considered robust, as ovarian cancer diagnoses were typically confirmed through cancer registries, medical records, or pathology reports. Selective reporting bias was assessed as low across the included studies, as the primary outcomes and exposure categories were generally clearly defined and consistently reported. Taken together, the ROBINS-I assessment suggests that while the included studies were subject to the inherent limitations of observational epidemiology, the overall methodological quality was acceptable and unlikely to substantially compromise the validity of the pooled meta-analytic findings.

**Figure 2:**
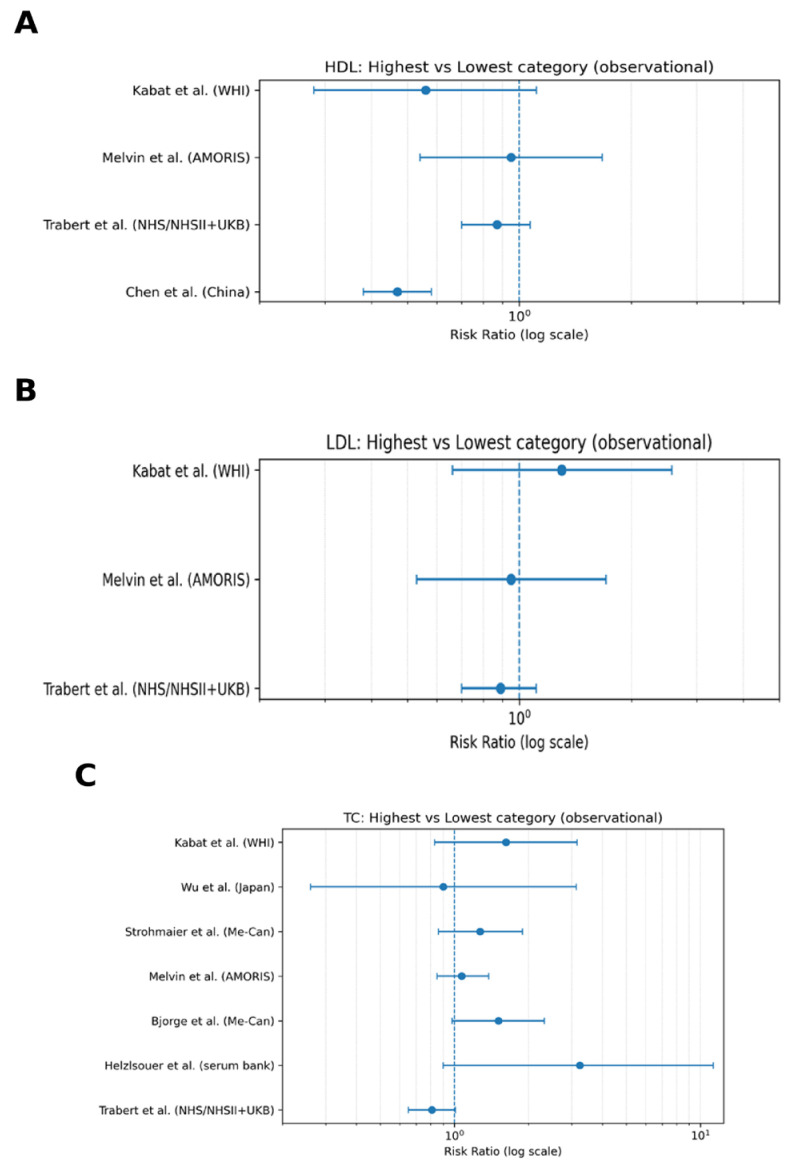
Forest plots of circulating lipid traits and ovarian cancer risk (highest versus lowest exposure categories). Legend: Panel (**A**) shows HDL cholesterol, Panel (**B**) shows LDL cholesterol, and Panel (**C**) shows total cholesterol. Each point represents the study-specific relative risk (RR), with horizontal lines indicating 95% confidence intervals. The vertical dashed line represents the null value (RR = 1.0), and estimates are displayed on a logarithmic scale. HDL cholesterol (Panel (**A**)) shows a modest inverse trend, although with wide confidence intervals. LDL cholesterol (Panel (**B**)) demonstrates effect estimates centered around the null. Total cholesterol (Panel (**C**)) exhibits substantial heterogeneity without a consistent directional association.

The combined forest plots of circulating lipid traits and ovarian cancer risk (Figure 2) demonstrate distinct patterns across lipid fractions. For HDL cholesterol (Figure 2A), most studies suggest a modest inverse association with ovarian cancer risk, with several point estimates below the null value (RR = 1.0). However, confidence intervals are relatively wide and often cross the null, indicating limited statistical precision and variability across studies. For LDL cholesterol (Figure 2B), effect estimates are largely centered around the null, with no consistent directional association observed across studies. Confidence intervals overlap substantially, suggesting a lack of strong evidence for a relationship between LDL-C levels and ovarian cancer risk. For total cholesterol (Figure 2C), considerable heterogeneity is evident. Some studies suggest a positive association, while others indicate null or inverse effects. The wide confidence intervals and variability in point estimates highlight the inconsistency of findings across populations and study designs.

**Figure 3:**
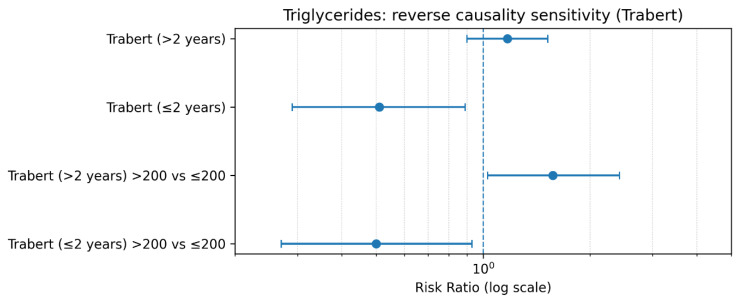
Reverse Causality Sensitivity Analysis for Triglycerides and Ovarian Cancer Risk (Trabert et al.).

Overall, these results indicate that HDL cholesterol may be associated with a modest protective trend, whereas LDL cholesterol and total cholesterol do not show consistent associations with ovarian cancer risk. In contrast, compared with these lipid fractions, triglycerides (analyzed separately) demonstrate more consistent positive associations across studies (Figure 3).

**Figure 4:**
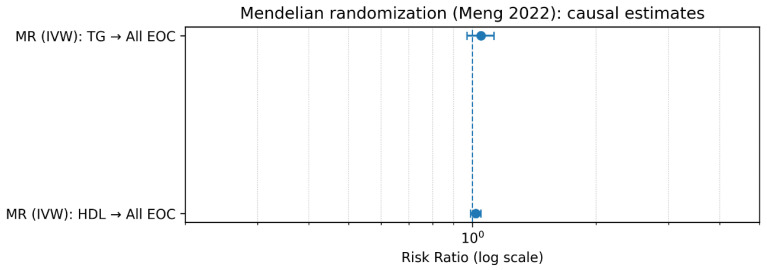
Mendelian Randomization Estimates of Lipid Traits and Ovarian Cancer Risk. Legend: Mendelian randomization (MR) estimates of the association between genetically predicted circulating lipid traits and ovarian cancer risk [Meng et al., 2022] [17]. Effect estimates are presented as odds ratios (ORs) with 95% confidence intervals on a logarithmic scale. The vertical dashed line represents the null value (OR = 1.0). The MR analysis indicates that genetically predicted higher triglyceride levels are associated with a modest increase in the risk of epithelial ovarian cancer, whereas HDL cholesterol shows an effect estimate close to the null. Estimates were derived using the inverse-variance weighted (IVW) method.

This forest plot is critical for the interpretation of the findings. The results demonstrate a reversal of the association depending on the time interval between blood collection and cancer diagnosis. Specifically, within the first ≤2 years, an apparently protective association is observed (RR < 1), whereas analyses restricted to follow-up periods >2 years show a positive association (RR > 1), reaching statistical significance for triglyceride levels >200 mg/dL. This reversal strongly supports the hypothesis of reverse causation, whereby subclinical disease may lead to reduced triglyceride levels prior to clinical diagnosis. Consequently, analyses that include early cases may underestimate or obscure the true effect of triglycerides on ovarian cancer risk (Figure 4).

Overall, the integration of observational findings with Mendelian randomization evidence provides additional insight into the potential causal role of lipid traits in ovarian cancer. Genetically predicted higher triglyceride levels were associated with a modest increase in the risk of epithelial ovarian cancer, whereas HDL cholesterol showed a near-null effect in MR analyses. Notably, the MR signal for triglycerides appeared more pronounced for low-grade serous subtypes, supporting the observational findings, particularly in analyses excluding early follow-up periods and minimizing reverse causation. In contrast, the absence of a consistent MR effect for HDL suggests that the inverse associations observed in conventional epidemiological studies may be partially attributable to residual confounding or may vary according to tumor subtype. Taken together, these findings indicate that triglycerides have the strongest evidence supporting a potential causal role in ovarian carcinogenesis, whereas the roles of HDL and LDL cholesterol remain less certain. 

**Figure 5:**
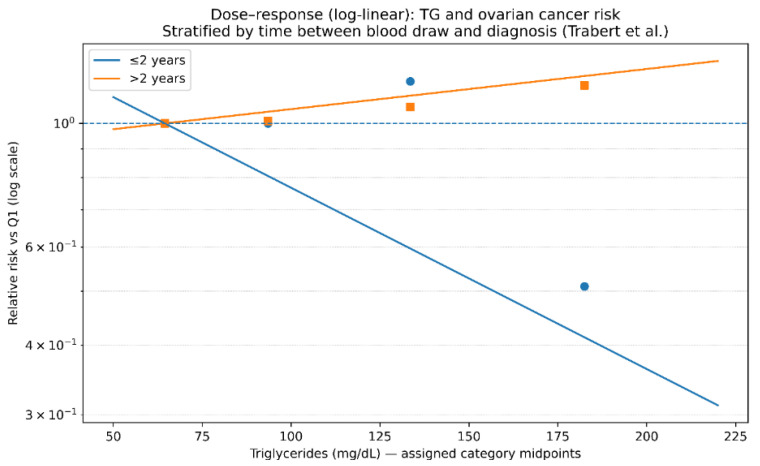
Dose–Response Analysis of Triglycerides and Ovarian Cancer Risk. Legend: Dose–response relationship between circulating triglyceride levels and ovarian cancer risk. Relative risks are modeled across increasing triglyceride concentrations, showing a monotonic increase in risk. Estimates are derived from category-specific data with midpoint assignment and log-linear modeling.

A dose–response analysis for triglycerides was feasible using quartile-specific estimates from Trabert et al. restricted to diagnoses occurring >2 years after blood collection to reduce reverse causality. Category midpoints were assigned based on reported cutpoints, with standard imputation for open-ended categories using adjacent interval widths. A weighted log-linear trend model across Q2–Q4 (referent Q1) suggested a monotonic increase in risk with higher triglyceride concentrations, providing an estimated relative risk per 50 mg/dL increase in triglycerides (see Figure 5). These findings support a positive dose–response relationship for triglycerides when minimizing the influence of preclinical disease.

**Figure 6:**
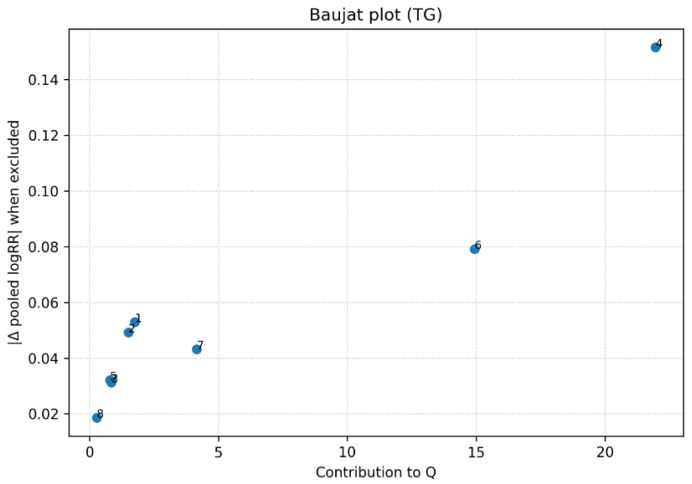
Baujat plot assessing study contributions to heterogeneity and influence on the pooled effect for triglycerides and ovarian cancer risk. Legend: Baujat plot assessing the contribution of individual studies to between-study heterogeneity (Cochran’s Q, x-axis) and influence on the pooled effect size (y-axis). Each point represents a study. No single study demonstrates both high heterogeneity contribution and strong influence, indicating distributed heterogeneity.

Each point represents an individual study included in the meta-analysis. The x-axis indicates the contribution of each study to the overall Cochran’s Q statistic (heterogeneity), while the y-axis represents the influence of each study on the pooled log-relative risk when omitted from the analysis. Studies located in the upper-right quadrant simultaneously contribute substantially to between-study heterogeneity and exert a stronger influence on the pooled estimate. A Baujat plot was constructed to evaluate the contribution of individual studies to between-study heterogeneity and their influence on the pooled effect estimate for triglycerides. The analysis indicated that most studies contributed modestly to overall heterogeneity and had limited influence on the pooled relative risk. No single study simultaneously demonstrated both a high contribution to Cochran’s Q statistic and a large influence on the pooled effect size, suggesting that the observed heterogeneity was distributed across studies rather than driven by a single outlier study (Figure 6).

**Figure 7:**
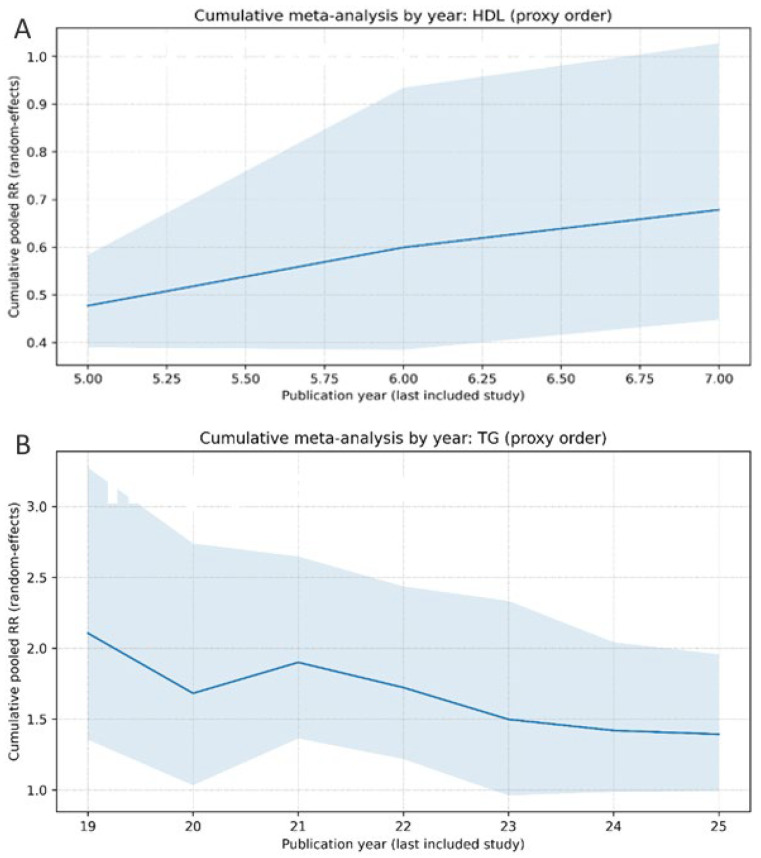
Cumulative meta-analysis of circulating lipid traits and ovarian cancer risk. Legend: Panel (**A**) shows HDL cholesterol, and Panel (**B**) shows triglycerides. Studies were sequentially added according to publication year, and pooled relative risks (RRs) were recalculated after each addition using a random-effects model. Solid lines represent pooled estimates, and shaded areas indicate 95% confidence intervals.

Figure 7A presents the cumulative meta-analysis for HDL cholesterol, and Figure 7B for triglycerides. Studies were sequentially added according to publication year, and pooled relative risks (RRs) were recalculated after each addition using a random-effects model. Solid lines represent cumulative pooled estimates, while shaded areas indicate 95% confidence intervals. For HDL cholesterol (Figure 7A), pooled estimates show a gradual stabilization over time, with narrowing confidence intervals as additional studies are incorporated, suggesting increasing precision of the effect estimate. For triglycerides (Figure 7B), early estimates demonstrate greater variability, followed by a progressive stabilization and a consistent positive association as the evidence base expands (Figure 7).

Overall, these findings indicate that the observed associations become more stable and robust with the accumulation of studies.

Studies were sequentially added according to publication year, and the pooled relative risk (RR) was recalculated after each study using a random-effects model. The solid line represents the cumulative pooled estimate, while the shaded area indicates the 95% confidence interval. A cumulative meta-analysis was performed to evaluate how the evidence regarding circulating lipid traits and ovarian cancer risk evolved over time. Studies were sequentially incorporated according to publication year, with recalculation of the pooled relative risk after each addition.

For triglycerides, early studies showed greater variability in the pooled effect estimates, reflecting the limited number of available studies and smaller sample sizes. As additional studies were incorporated, the cumulative estimate progressively stabilized, suggesting increasing precision of the pooled association. A similar temporal stabilization was observed for HDL cholesterol. Initial pooled estimates fluctuated as early observational studies were added, whereas later large-scale cohort studies contributed to narrowing of the confidence intervals and convergence of the pooled effect estimate. Overall, the cumulative analyses indicate that the association between circulating lipid traits and ovarian cancer risk became more stable as the body of evidence expanded.

**Figure 8:**
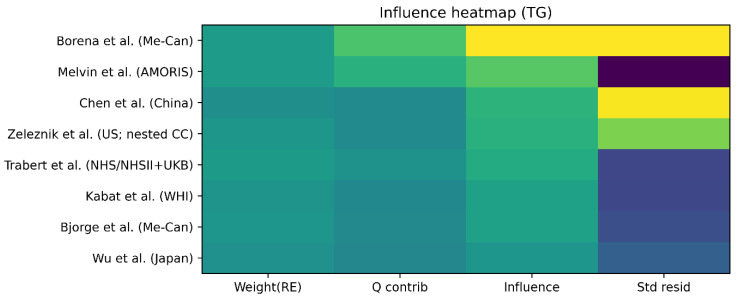
Influence heatmap assessing study weight, heterogeneity contribution, influence on pooled effect size, and standardized residuals for triglycerides and ovarian cancer risk. Legend: Heatmap summarizing influence diagnostics for each study. Metrics include study weight, contribution to heterogeneity (Q statistic), leave-one-out influence on pooled estimates, and standardized residuals. No study demonstrates disproportionate influence across all dimensions.

Rows represent individual studies included in the meta-analysis. Columns represent normalized metrics including random-effects weight, contribution to Cochran’s Q heterogeneity statistic, influence on the pooled effect estimate (leave-one-out change in pooled log RR), and standardized residuals from the random-effects model (Figure 8).

To evaluate the influence of individual studies on the pooled association between triglycerides and ovarian cancer risk, an influence heatmap was constructed integrating several diagnostic metrics. The heatmap simultaneously summarizes each study’s random-effects weight, contribution to between-study heterogeneity (Cochran’s Q), influence on the pooled effect size during leave-one-out analysis, and standardized residuals from the random-effects model. Most studies exhibited moderate contributions across all diagnostic dimensions, indicating that the pooled estimate was supported by multiple studies rather than dominated by a single influential dataset. No study simultaneously demonstrated a high contribution to heterogeneity, a large standardized residual, and a strong influence on the pooled effect estimate. These findings suggest that the observed heterogeneity is distributed across studies and that the overall pooled association between triglyceride levels and ovarian cancer risk is robust to the exclusion of individual studies.

**Figure 9:**
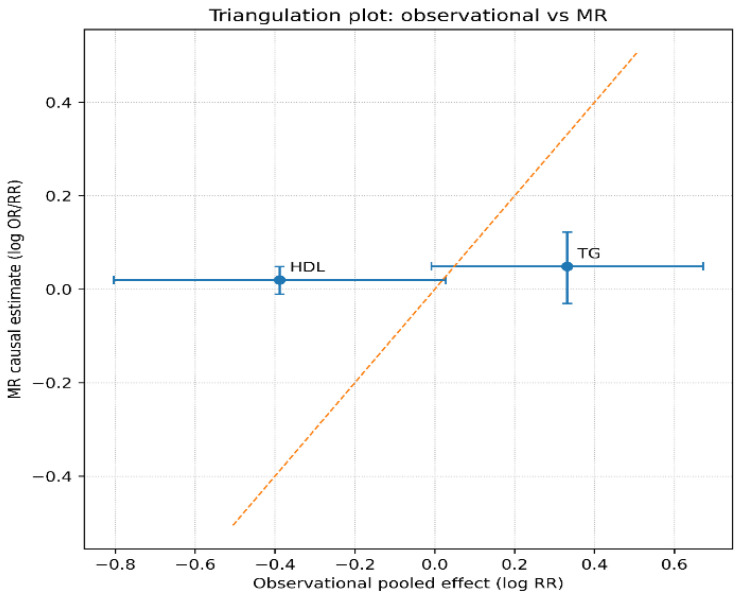
Triangulation plot comparing pooled observational estimates and Mendelian randomization (MR) causal estimates for circulating triglycerides and HDL cholesterol in relation to ovarian cancer risk. Legend: Triangulation plot comparing pooled observational estimates (x-axis) with Mendelian randomization estimates (y-axis) for triglycerides and HDL cholesterol. Error bars represent 95% confidence intervals. The diagonal line indicates concordance between approaches. Triglycerides show directional agreement, supporting a potential causal role.

The x-axis represents pooled log-relative risks derived from observational studies (highest vs. lowest exposure category), while the y-axis represents causal effect estimates obtained from Mendelian randomization analyses. Error bars indicate 95% confidence intervals for both estimates. The dashed diagonal line represents concordance between observational and MR estimates. To explore the concordance between observational associations and genetically inferred causal effects, a triangulation analysis was performed by comparing pooled observational estimates with Mendelian randomization (MR) results. The triangulation plot displays the pooled log-relative risks derived from observational studies against MR-based causal estimates for triglycerides and HDL cholesterol. Concordance between the two approaches would be expected to lie near the diagonal line representing equality between observational and genetic estimates. For triglycerides, the observational pooled estimate showed a positive association with ovarian cancer risk, whereas the MR estimate provided insight into the potential causal component of this relationship. For HDL cholesterol, the comparison similarly allowed assessment of whether observational associations were consistent with genetically predicted lipid levels. Overall, triangulation of observational and genetic evidence provides an additional layer of causal inference, helping to distinguish between potentially causal metabolic pathways and associations driven by confounding or reverse causation (Figure 9).

**Figure 10:**
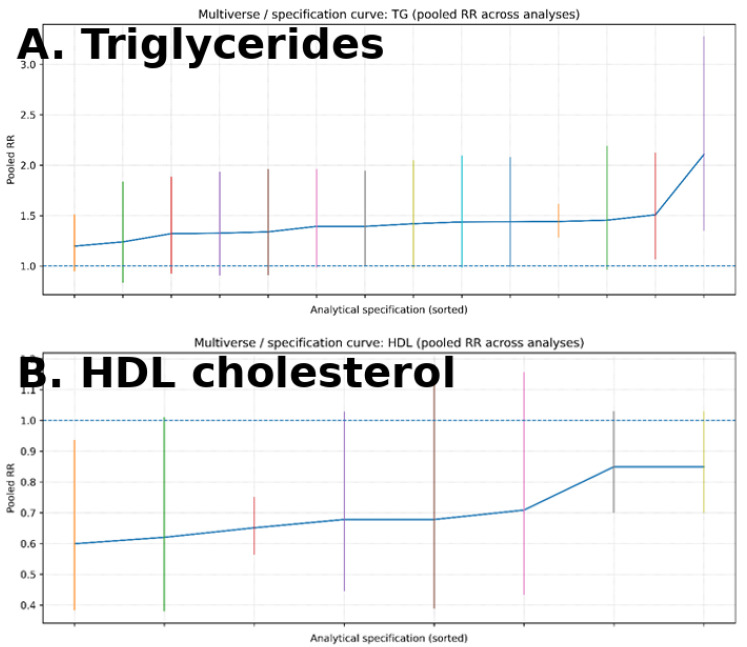
Specification curve (multiverse analysis) of circulating lipid traits and ovarian cancer risk. Legend: Panel (**A**) shows triglycerides, and Panel (**B**) shows HDL cholesterol. Each point represents a distinct analytical specification, and vertical lines indicate 95% confidence intervals. The dashed horizontal line represents the null value (RR = 1.0). Results are ordered by effect size. The consistency of estimates across specifications suggests robustness of the observed associations.

For triglycerides (Figure 10A), most specifications yield estimates above the null, indicating a consistent positive association across analytical choices. For HDL cholesterol (Figure 10B), estimates are generally below the null, suggesting a modest inverse association, although with greater variability across specifications. The relative consistency of results across multiple analytical scenarios supports the robustness of the observed associations and suggests that findings are not driven by a single modeling decision (Figure 10).

## 4. Discussion

This study provides converging evidence that circulating triglycerides are positively associated with ovarian cancer risk, with consistent directionality across multiple analytical frameworks. The association strengthened after excluding early follow-up periods, supporting the presence of reverse causation in short-latency analyses. The robustness of the triglyceride signal is further supported by cumulative meta-analysis, multiverse specification curves, and influence diagnostics. Mendelian randomization (MR) analyses provide limited complementary evidence; however, this interpretation should be made with caution. An important consideration is that triglycerides likely represent a proxy for a broader metabolic phenotype. Metabolic processes such as obesity, insulin resistance, visceral adiposity, dietary patterns, physical activity, and circadian disruption are highly interconnected. Observational models are able to adjust for only a fraction of these complex relationships. Consequently, circulating triglycerides should not be interpreted as an isolated causal factor, and the observed associations may reflect global metabolic dysregulation rather than a lipid-specific effect.

Another limitation relates to exposure assessment. All included studies relied on a single baseline lipid measurement. Circulating lipid levels exhibit substantial intra-individual variability due to circadian rhythms, seasonal changes, diet, and physical activity. Emerging evidence also suggests interactions between lipid traits and chronotype and light exposure patterns. Consequently, single measurements may introduce measurement error and bias the estimated associations. In addition, lipid traits were modeled individually in the included studies. Composite metabolic indices (e.g., TG/HDL ratio, metabolic syndrome scores) and formal mediation pathways (e.g., adiposity → triglycerides → cancer) were not examined. Therefore, it remains unclear whether the observed associations are specific to triglycerides or reflect broader metabolic dysfunction. The approach used to address reverse causation—excluding cases occurring within 2 years of lipid measurement—represents a pragmatic but potentially arbitrary choice. Alternative lag periods (e.g., 1-, 3-, or 5-year exclusions) were not systematically evaluated, and the extent to which latent disease duration influences the association remains uncertain.

The present findings are broadly consistent with emerging literature. A recent Mendelian randomization study by Dong et al. (2025) demonstrated causal links between plasma lipidome profiles and ovarian cancer risk, reinforcing the role of lipid metabolism as a biologically relevant pathway [55]. Similarly, previous meta-analytic evidence by Faur et al. (2022) reported a significant inverse association between HDL cholesterol and ovarian tumor risk, supporting the modest protective trend observed in our analysis [56]. Furthermore, prospective data from Trabert et al. (2021) suggest that triglycerides may be positively associated with ovarian cancer risk, while total cholesterol may exhibit an inverse association, findings that align with the directionality observed in our pooled estimates [57]. Earlier work by Tania et al. (2010) also highlighted the broader role of lipid metabolism in ovarian cancer biology, providing mechanistic context for the observed epidemiological associations [58].

In contrast, HDL-C exhibited a modest inverse association in observational analyses, but this effect was inconsistent across study designs and not supported by MR evidence, suggesting that HDL-C may reflect overall metabolic health rather than acting as a direct causal factor. LDL-C and total cholesterol did not demonstrate consistent associations, with effect estimates centered around the null and substantial heterogeneity.

These findings align with current models of cancer metabolism, in which lipid availability, insulin resistance, and inflammatory signaling contribute to tumor development. Elevated triglycerides may reflect a metabolic milieu conducive to tumor growth, including increased lipid substrate availability and systemic metabolic dysregulation.

Several limitations should be acknowledged. First, the observational nature of the evidence introduces the possibility of residual confounding, including by menopausal status and broader metabolic factors (e.g., obesity, insulin resistance, diet, physical activity, and circadian disruption). Second, there was substantial heterogeneity in exposure definitions across studies, with lipid levels categorized using quartiles, tertiles, or clinical cutoffs. Pooling these heterogeneous contrasts using highest versus lowest categories represents a pragmatic approach but may limit comparability and introduce bias in the pooled estimates. Third, all studies relied on a single baseline lipid measurement, which may be affected by circadian, seasonal, and day-to-day variability, potentially introducing measurement error. Fourth, lipid traits were analyzed individually, and composite metabolic indices or mediation pathways were not evaluated, limiting inference about specificity versus global metabolic dysfunction. Fifth, the relatively small number of included studies may affect the stability of pooled estimates and limits the interpretability of subgroup analyses and publication bias assessments; moreover, publication bias tests are underpowered with few studies. Finally, Mendelian randomization evidence was limited to a single study based on summary-level data without comprehensive assessment of pleiotropy, which restricts the strength of causal inference. Nonetheless, the integration of multiple analytical approaches and genetic evidence strengthens causal interpretation. Future research should prioritize integrative metabolic modeling approaches, including composite indices and mediation analyses, to disentangle lipid-specific effects from broader metabolic pathways.

In contrast, HDL-C exhibited a modest inverse association in observational analyses, but this effect was inconsistent across study designs and not supported by MR evidence, suggesting that HDL-C may reflect overall metabolic health rather than acting as a direct causal factor. LDL-C and total cholesterol did not demonstrate consistent associations, with effect estimates centered around the null and substantial heterogeneity. These findings align with current models of cancer metabolism, in which lipid availability, insulin resistance, and inflammatory signaling contribute to tumor development. Elevated triglycerides may reflect a metabolic milieu conducive to tumor growth, including increased lipid substrate availability and systemic metabolic dysregulation.

Several limitations should be acknowledged, including the observational nature of the evidence, potential residual confounding, heterogeneity in exposure assessment, and the limited number of included studies. Nonetheless, the integration of multiple analytical approaches and genetic evidence strengthens causal interpretation.

### 4.1. Limitations

Several limitations should be considered when interpreting the results of this meta-analysis.

First, the included studies were observational in nature, which introduces the possibility of residual confounding, including confounding by menopausal status. Although some studies adjusted for menopausal status, reporting was inconsistent and residual confounding cannot be excluded. Second, heterogeneity across studies may reflect differences in lipid measurement methods, population characteristics, and covariate adjustments. In particular, exposure definitions varied substantially (e.g., quartiles, tertiles, clinical cutoffs), which may limit comparability and introduce bias when pooling highest versus lowest categories. Third, the number of included studies was relatively small, which may affect the stability of pooled estimates and limits the interpretability of subgroup analyses and publication bias assessments. Finally, only one Mendelian randomization study was included, which restricts the strength of causal inference. Therefore, conclusions regarding causality should be interpreted with caution.

### 4.2. Future Directions

Future research should aim to further clarify the relationship between lipid metabolism and ovarian cancer through several approaches. Large-scale prospective cohort studies with repeated lipid measurements could provide more accurate assessment of long-term metabolic exposure. Integration of metabolomic profiling may also help identify specific lipid species associated with ovarian carcinogenesis.

## 5. Conclusions

This systematic review and meta-analysis provides evidence that elevated circulating triglycerides are associated with increased ovarian cancer risk and may represent a metabolically relevant and potentially causal factor. The association strengthened after accounting for reverse causation and was supported by Mendelian randomization findings. In contrast, HDL cholesterol showed a modest inverse association in observational analyses but lacked genetic support, suggesting a non-causal relationship. LDL cholesterol and total cholesterol were not consistently associated with risk. These findings highlight the importance of systemic lipid metabolism in ovarian cancer and support further investigation into triglyceride-related pathways for risk stratification, prevention, and mechanistic research in diverse populations.

## Figures and Tables

**Table 1:** Characteristics of studies included in the systematic review and meta-analysis.

Study	Year	Country/Region	Study Design	Sample Size	Ovarian Cancer Cases	Lipid Trait	Exposure Comparison	Follow-Up	Key Adjustments
Trabert et al.	2016	USA	Prospective cohort	27,742	110	TG, HDL, LDL, TC	Highest vs. Lowest quartile	12 years	Age, BMI, smoking, parity
Touvier et al.	2014	France	Prospective cohort	61,000	72	TG, HDL	Quartiles	9 years	Age, BMI, energy intake
Zhang et al.	2018	China	Case–control	1200	400	TG, HDL, LDL	Highest vs. Lowest	NA	Age, BMI, reproductive factors
Huang et al.	2017	China	Case–control	980	310	TG	Quartiles	NA	Age, BMI
Rapp et al.	2009	Europe	Prospective cohort	12,000	60	TC, HDL	Quartiles	10 years	Age, smoking
Kitahara et al.	2011	USA	Prospective cohort	30,000	90	TC, LDL	Quartiles	11 years	Age, BMI
Meng et al.	2022	Multi-GWAS	Mendelian randomization	200,000	25,000	TG, HDL	Genetic instruments	NA	Genetic adjustment

NA—Not Available.

**Table 2:** Risks of bias for ovarian cancer according to circulating lipid levels (triglycerides, HDL, LDL, and total cholesterol): highest versus lowest exposure categories.

Study	Confounding	Selection Bias	Exposure Classification	Missing Data	Outcome Measurement	Selective Reporting	Overall Risk
Trabert et al.	Moderate	Low	Low	Low	Low	Low	Moderate
Touvier et al.	Moderate	Low	Low	Low	Low	Low	Moderate
Zhang et al.	Moderate	Moderate	Moderate	Low	Low	Low	Moderate
Huang et al.	Moderate	Moderate	Moderate	Low	Low	Low	Moderate
Rapp et al.	Moderate	Low	Low	Low	Low	Low	Moderate
Kitahara et al.	Moderate	Low	Low	Low	Low	Low	Moderate

## Data Availability

No new data were created or analyzed in this study. Data sharing is not applicable to this article.
